# Osthole Alleviates Neointimal Hyperplasia in Balloon-Induced Arterial Wall Injury by Suppressing Vascular Smooth Muscle Cell Proliferation and Downregulating Cyclin D1/CDK4 and Cyclin E1/CDK2 Expression

**DOI:** 10.3389/fphys.2020.514494

**Published:** 2021-01-26

**Authors:** Yi-Qi Li, Ye-Li Li, Xiao-Tong Li, Jun-Yuan Lv, Yang Gao, Wen-Na Li, Qi-Hai Gong, Dan-Li Yang

**Affiliations:** ^1^Joint International Research Laboratory of Ethnomedicine of Ministry of Education, Key Laboratory of Basic Pharmacology of the Ministry of Education, The Key Laboratory of Basic Pharmacology of Guizhou Province, Department of Pharmacology, School of Pharmacy, Zunyi Medical University, Zunyi, China; ^2^Department of Pharmacology, Zhuhai Campus of Zunyi Medical University, Zhuhai, China; ^3^Department of Breast and Thyroid Surgery, Affiliated Hospital of Zunyi Medical University, Zunyi, China

**Keywords:** osthole, platelet-derived growth factor-BB, vascular smooth muscle cells, proliferation, cyclin D1, CDK4, cyclin E1, CDK2

## Abstract

Percutaneous coronary intervention (PCI) is the most widely used therapy for treating ischemic heart disease. However, intimal hyperplasia and restenosis usually occur within months after angioplasty. Modern pharmacological researchers have proven that osthole, the major active coumarin of *Cnidium monnieri* (L.) Cusson, exerts potent antiproliferative effects in lung cancer cells, the human laryngeal cancer cell line RK33 and TE671 medulloblastoma cells, and its mechanism of action is related to cell cycle arrest. The goal of the present study was to observe the effect of osthole on vascular smooth muscle cell (VSMC) proliferation using platelet-derived growth factor-BB (PDGF-BB)-stimulated VSMCs isolated from rats and vascular balloon injury as models to further elucidate the molecular mechanisms underlying this activity. We detected the relative number of VSMCs by the MTT assay and EdU staining and examined cell cycle progression by flow cytometry. To more deeply probe the mechanisms, the protein expression levels of PCNA, the cyclin D1/CDK4 complex and the cyclin E1/CDK2 complex in balloon-treated rat carotid arteries and the mRNA and protein expression levels of the cyclin D1/CDK4 and cyclin E1/CDK2 complexes in VSMCs were detected by real-time RT-PCR and western blotting. The data showed that osthole significantly inhibited the proliferation of VSMCs induced by PDGF-BB. Furthermore, osthole caused apparent VSMC cycle arrest early in G0/G1 phase and decreased the expression of cyclin D1/CDK4 and cyclin E1/CDK2. Our results demonstrate that osthole can significantly inhibit PDGF-BB-induced VSMC proliferation and that its regulatory effects on cell cycle progression and proliferation may be related to the downregulation of cyclin D1/CDK4 and cyclin E1/CDK2 expression as well as the prevention of cell cycle progression from G0/G1 phase to S phase. The abovementioned mechanism may be responsible for the alleviation of neointimal hyperplasia in balloon-induced arterial wall injury by osthole.

## Introduction

Percutaneous coronary intervention (PCI) is the most widely used therapy for treating ischemic heart disease. However, intimal hyperplasia and restenosis usually occur within months after angioplasty. Many studies have shown that the hallmark of intimal hyperplasia is the excessive proliferation of vascular smooth muscle cells (VSMCs), which is an early response to the arterial wall injury (Direnzo et al., 2016; Tang and Gerlach, 2017). In their quiescent state, these cells help to modulate the blood supply *via* vasoconstriction and vasodilatation. Upon activation, VSMCs proliferate at a very high rate and migrate from the media into the lumen of blood vessels (Qiu et al., 2013); meanwhile, a large number of cytokines and growth factors released from activated cells also contribute to this progression (Liu et al., 2009, 2013). Therefore, inhibiting VSMC proliferation may represent a potential therapeutic target for preventing and treating vascular proliferative diseases.

Cell proliferation occurs through the regulation of the cell cycle, a complex and tightly controlled process. The G0, G1, S, G2, and M phases are five sequential stages involved in this process. The correct progression of the cell cycle is guaranteed because activation of each phase is dependent on the successful completion of previous stages (Begum et al., 2011). Molecular analysis of the cell cycle has shown that regulatory molecules (cyclins) and catalytic molecules (cyclin-dependent kinases, CDKs) act as the master regulators in this process and determine whether a cell commits to division or leaves the cell cycle (Urrego et al., 2014). The cyclin D1/CDK4 complex is required to promote the progression of cells from G0/G1 phase to S phase. The inhibition of cyclin D1 can arrest cells in G0/G1 phase (Liu et al., 2009).

*Cnidium monnieri* (L.) Cusson has been used as a traditional Chinese medicine for the treatment of gynecological diseases, cutaneous pruritus, and nephritis (Li et al., 2015; Zhang et al., 2015). Osthole (7-methoxy-8-isopentenoxy-coumarin), one of the pharmacologically active coumarins of *Cnidium monnieri* (L.) Cusson, has been reported to have cardioprotective (Duan et al., 2016), anti-inflammatory (Li et al., 2014), and antihypertensive activities (Fusia et al., 2012). Previous studies demonstrated that osthole suppresses the proliferation of pulmonary arterial smooth muscle cells (Yue et al., 2020), lung cancer cells (Xu et al., 2011), the human laryngeal cancer cell line RK33, and TE671 medulloblastoma cells (Jarzab et al., 2014); specifically, the associated mechanism is related to cell cycle arrest. Interestingly, data have indicated that some characteristics of cardiovascular diseases, such as atherosclerosis and angiostenosis (Jiang et al., 2013), are similar to those of cancer. In our previous study, we found that inflammatory mediators affect the rat carotid artery after balloon injury and the antiproliferative effect of osthole are related to the NF-*κ*B and TGF-*β*1/Smad2 signaling pathways (Li et al., 2017). Despite these observations, the exact mechanisms still require further investigation. In the present study, we aimed to observe the effect of osthole on VSMC proliferation using PDGF-BB-stimulated VSMCs isolated from rats and vascular balloon injury as models to further elucidate the molecular mechanisms underlying this activity.

## Materials and Methods

### Animals and Materials

Male Sprague-Dawley rats were obtained from the Animal Center of the Research Institute of Surgery at the Third Military Medical University (Chongqing, China). Osthole (purity 98.0%) was purchased from Nanjing Zelang Medical Technology Co., Ltd. (Nanjing, China), dissolved to 160mM (in dimethyl sulfoxide, DMSO) as a stock solution and stored at −20°C. Platelet-derived growth factor-BB (PDGF-BB) was purchased from Sigma-Aldrich Co (United States). A Cell Cycle Assay Kit was obtained from KeyGen BioTech (Nanjing, China). 2-F Fogarty balloon catheters were purchased from Edwards Lifesciences Co (United States). MTT (3-(4,5-dimethylthiazol-2-yl)-2,5- diphenyltetrazolium bromide) was obtained from Sigma Chemical Co (St. Louis, MO, United States). A mouse anti-rat monoclonal PCNA antibody and rabbit anti-rat polyclonal antibodies targeting cyclin D1, cyclin E1, CDK2, and CDK4 were purchased from Abcam Co (United States). The primers used in the study were designed and synthesized by TaKaRa Systems (Dalian, China), and IQ™ SYBR® Green Supermix was obtained from BIO-RAD (United States).

### Vascular Balloon Injury Model

For balloon injury, 8-week-old male Sprague-Dawley rats (320 ± 20g) were anesthetized *via* intraperitoneal administration of sodium pentobarbital (40mg/kg), after which the left external carotid arteries were exposed, and a 2-F Fogarty balloon catheter was introduced into the left common carotid artery. The catheter was inflated and passed through the artery three times to ensure complete and reproducible removal of the endothelial lining. Finally, the wound was ligated and closed following the last passage of the balloon. In the sham group, animals were subjected to left external carotid artery exposure, but a balloon catheter was not inserted into the vessel. All animal experiments were performed in accordance with Animal Care and Use Guidelines of China and were approved by the Animal Use and Care Committee of Zunyi Medical University.

### Arterial Harvest and Histological Examination

The rats were randomly divided into the following four groups (*n* = 6): the sham group, model group, 20mg/kg/d osthole-treated (Osthole-20) group, and 40mg/kg/d osthole-treated (Osthole-40) group. All treatments were administered daily *via* oral gavage on the first day following surgery. The rats in the sham and model groups were given normal saline, which is also used as vehicle in Osthole-20 and Osthole-40 groups. Fourteen days after balloon injury, the rats were euthanatized to detect vascular injury and intimal thickening of the target arteries. The left common carotid arteries were harvested and fixed with a 4% formaldehyde solution. The tissues were embedded in paraffin, sectioned at a thickness of 4μm, and stained with hematoxylin and eosin (H&E). The neointimal thickness and the ratio of tunica intima/media were analyzed using computer-assisted morphometry.

### Vascular Smooth Muscle Cell Culture and Experimental Design

VSMCs were isolated from the thoracic aortas of 8-week-old male Sprague-Dawley rats (180 ± 20g). The aortas were stripped of their endothelium and adventitia and were cut into small pieces, which were placed in cell culture flask containing Dulbecco’s modified Eagle’s medium supplemented with 20% fetal bovine serum and incubated at 37°C in a CO_2_ incubator (95% air and 5% CO_2_) for 10days. The medium was changed every 3days, and cells were removed by trypsinization and successively subcultured. Subcultured VSMCs between passages 3 and 6 were used for this experiment.

The cultured VSMCs were randomly divided into six groups: control group (control), no drug was added; DMSO group (DMSO), 0.5% DMSO was added in normal cells; PDGF-BB group: PDGF-BB 25ng/ml was added in normal grown cells; osthole low-dose group (Osthole-20), middle-dose group (Osthole-40), and high-dose group (Osthole-80): osthole 20, 40, and 80μM were added, respectively, to the cells treated with 25ng/ml PDGF-BB (PDGF-BB was added at 60min before the addition of osthole). The above concentrations of PDGF-BB or osthole used in each group were final concentration.

### Assessment of VSMC Proliferation by the MTT Assay

Rat VSMCs were plated in 96-well plates at a density of 8 × 10^4^ cells/ml and treated with PDGF-BB prior to osthole (PDGF-BB was added at 60min before the addition of osthole). After the cells were cultured for 24h, MTT assays were carried out as follows: VSMCs were grown in 100μl of medium at 37°C under 5% CO_2_ for 24h, after which 10μl of a 3-(4,5-dimethylthiazol-2-yl)-2,5-diphenyltetrazolium bromide (MTT) solution was added to each well for 4h to allow MTT reduction. The resulting formazan crystals were dissolved in DMSO, and the absorbance values were measured at a wavelength of 490nm on a microplate reader. Each condition was conducted in triplicate.

### EdU Staining

Cell proliferation was analyzed using a Cell-light EdU Apollo488 Kit according to the manufacturer’s protocol (RIBOBio. Co., Cat. No. C10310-3, Guangzhou, China). The logarithmic growth of VSMCs seeded in 96-well plates at a density of 8 × 10^4^ cells/ml was determined. Following treatment with PDGF-BB and osthole, the cells in each well were incubated with 50μM EdU for 3h. The cells were permeabilized for 10min with 0.5% Triton X-100 after being fixed in PBS containing 4% paraformaldehyde for 30min at room temperature. After washing with PBS, the cells were stained with Hoechst 33342 in the dark, washed, and imaged using a fluorescence microscope.

### Cell Cycle Analysis Using Flow Cytometry

VSMCs were treated with either PDGF-BB or different concentrations of osthole for 24h, and cell cycle distribution was determined by flow cytometry. Briefly, cells were harvested by trypsinization, washed twice in PBS, and fixed in 70% ice-cold ethanol overnight. Then, the ethanol was removed, and the cells were resuspended in PBS. After they were treated with 100μl RNase at 37°C for 30min, the cells were stained with 100μl of propidium iodide (PI) and incubated in the dark at 4°C. The cell cycle distribution was analyzed on a flow cytometer (Beckman Coulter, United States).

### Real-Time Quantitative Reverse Transcription-PCR

Total RNA was extracted using RNAiso Plus and then reverse-transcribed in a reaction containing PrimeScript™ RT Enzyme Mix, oligodT primer, random 6-mers, and PrimeScript™ Buffer (5×). To detect the mRNA expression levels of cyclin D1, cyclin E1, CDK2, and CDK4, we performed a two-step RT-PCR using an iCycler iQ Real-Time PCR Detection System with IQ™ SYBR®GREEN Supermix. The following primers were used for this experiment: cyclin D1 (GenBank accession no. NM_171992.4), forward primer: (5'–3') TACCGCACAACGCACTTTC and reverse primer: (5'–3') AAGGGCTTCAATCTGTTCCTG; cyclin E1 (GenBank accession no. NM_001100821.1), forward primer: (5'–3') TTTGCAAGATCCGGATGAA and reverse primer: (5'–3') CGCTGAATCATCATCCCAAG; CDK2 (GenBank accession no. NM_199501.1), forward primer: (5'–3') CCTGCACCAGGACCTCAAGAA and reverse primer: (5'–3') CGGTGAGAATGGCAGAATGCTA; CDK4 (GenBank accession no. NM_053593.2), forward primer: (5'–3') AGTCAGTGGTGCCGGAGATG and reverse primer: (5'–3') CAGCGTCCGGAAACTGGAA; and β-actin (GenBank accession no. NM_031144.2), forward primer: (5'–3') GGAGATTACT GCCCTGGCTCCTA and reverse primer: (5'–3') GACTCATCGTACTCCTGCTTGCTG. The threshold cycle (Ct) values of the target genes were normalized to those of β-actin in the same samples.

### Western Blot Analysis

Carotid arteries were pulverized with an electric homogenizer in cold RIPA lysis buffer containing proteinase inhibitors. The dissolved proteins were prepared by centrifugation at 12,000 × *g* for 10min at 4°C, after which the supernatant was collected. The protein concentrations were quantified by a Bicinchoninic Acid (BCA) Protein Assay Kit. After the VSMCs were subjected to their respective treatments for 24h, they were washed with ice-cold PBS and homogenized in a lysis buffer. The protein concentration was determined by a Bicinchoninic Acid (BCA) Protein Assay Kit. For western blot analysis, equal quantities of protein were separated on SDS-polyacrylamide gel electrophoresis (SDS-PAGE) gels. After electrophoresis, the proteins were electroblotted to polyvinylidene difluoride (PVDF) membranes and subsequently incubated in 5% nonfat dry milk in tris-buffered saline containing Tween (TBST) for 1h at room temperature. After the membranes were washed with TBST and incubated with anti-PCNA (1:1,000), anti-cyclin D1 (1:10,000), anti-cyclin E1 (1:1,000), anti-CDK2 (1:1,000), and anti-CDK4 (1:500) antibodies in 1% nonfat dry milk in TBST overnight at 4°C, they were washed with TBST for 10min three times and then incubated with horseradish peroxidase (HRP)-conjugated secondary antibodies for 2h at room temperature. The immunoblots were visualized *via* enhanced chemiluminescence (ECL-Plus; Beyotime, P0018, Shanghai, China), and the band intensities were quantified using Quantity One® software.

### Data Analysis

Data were analyzed using one-way ANOVA with SPSS 17.0 software, and all the results are presented as the mean ± S.E.M. *Post hoc* comparisons were performed using LSD with equal variances and with Dunnett’s T3 with unequal variances, *p* < 0.05 was considered statistically significant.

## Results

### Effects of Osthole on Neointimal Hyperplasia

**Figure 1:**
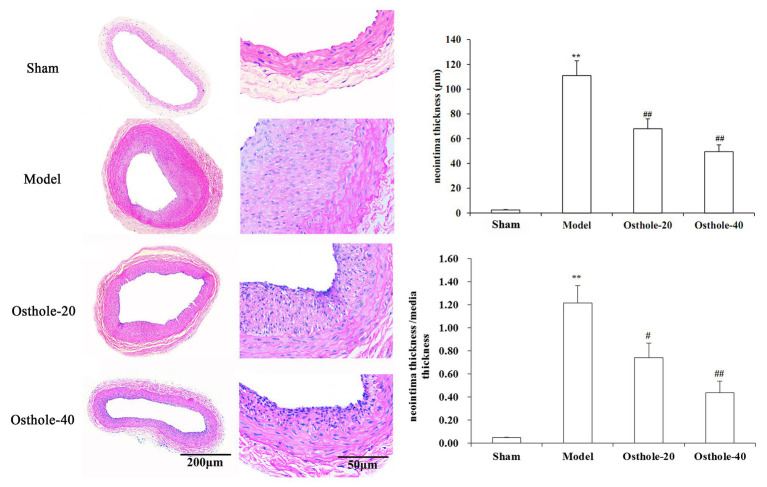
Effects of osthole on neointimal formation in rat carotid arteries. Representative sections from the sham, model, Osthole-20, and Osthole-40 groups. The neointimal thickness and the ratio of tunica intima/media of each group is expressed as the mean ± SEM. Data are presented as the mean ± SEM, *n* = 6. ^**^Significant difference compared to the sham group at *p* < 0.01, ^#^Significant difference compared to the model group at *p* < 0.05, ^##^Significant difference compared to the model group at *p* < 0.01.

Digital images were acquired with the Q Win image manipulation system. As shown in Figure 1, hematoxylin and eosin (H&E) staining showed that no neointima was found in the vessels not subjected to balloon angioplasty, and the model group showed bulky concentric neointimal hyperplasia as well as a disrupted elastic membrane after balloon injury (*p* < 0.01). However, The results from the image analysis system suggested that there was an obvious reduction in neointimal thickness and the ratio of tunica intima/media in balloon-injured sections from the osthole-treated animals, with 38% (Osthole-20) and 64% (Osthole-40) reductions in the neointimal thickness compared with that in the model group (*p* < 0.01).

### Effects of Osthole on PCNA, Cyclin D1, CDK4, Cyclin E1, and CDK2 Protein Expression Levels in Balloon-Treated Rat Carotid Arteries

**Figure 2:**
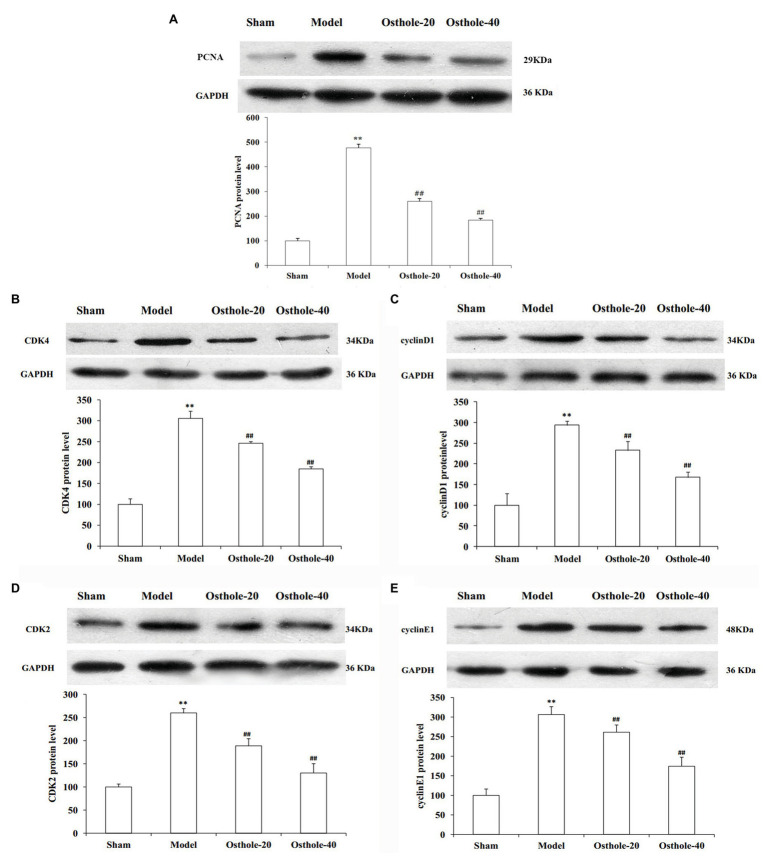
Effects of osthole on PCNA, cyclinD1, CDK4, cyclin E1, and CDK2 protein expression in balloon-treated rat carotid arteries. **(A)** PCNA protein expression. **(B)** CDK4 protein expression. **(C)** Cyclin D1 protein expression. **(D)** CDK2 protein expression. **(E)** Cyclin E1 protein expression. Data are presented as the mean ± S.E.M. *n* = 4. ^**^Significant difference compared with the sham group at *p* < 0.01, ^##^Significant difference compared with the model group at *p* < 0.01.

To understand the mechanisms underlying the protective effects of osthole on neointimal formation in rat carotid arteries, the protein expression of PCNA, cyclin D1, CDK4, cyclin E1, CDK2 in the carotid arteries was measured by western blotting. As shown in Figure 2, compared with that in the sham group, the protein expression of PCNA, cyclin D1, CDK4, cyclin E1, and CDK2 was significantly increased by 3.1, 2.9, 2.5, and 3.1 times, respectively (*p* < 0.01). However, compared with that in the model group, the expression of these proteins was decreased by 19, 20, 27, and 15% respectively, upon treatment with osthole at a dosage of 20mg/kg/day (*p* < 0.01), and the expression of these proteins was further downregulated by 40, 43, 50, and 43%, respectively, upon treatment with osthole at a dosage of 40mg/kg/day (*p* < 0.01).

### Antiproliferative Effect of Osthole on PDGF-BB-Stimulated VSMCs

**Figure 3:**
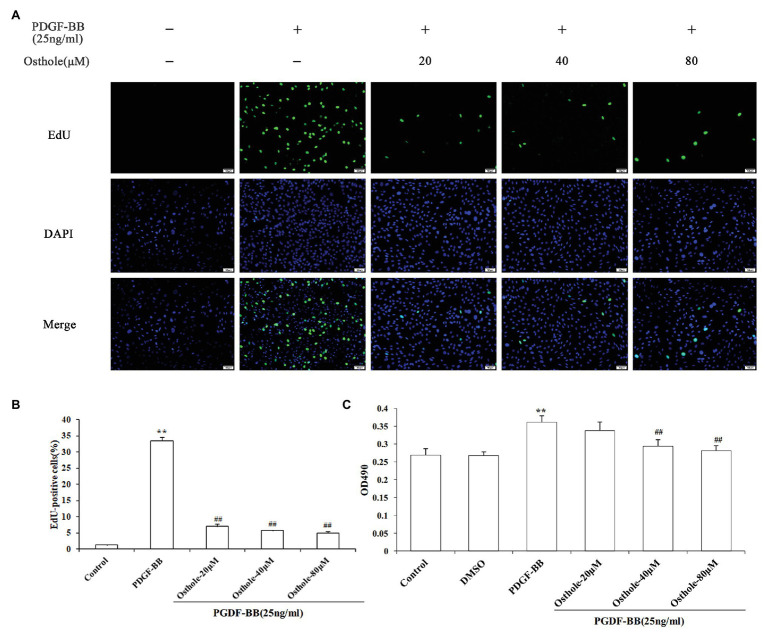
Cells were rendered quiescent and treated with PDGF-BB, after which osthole (20, 40, and 80μM) was added and incubated for 24h. **(A)** Fluorescence images of EdU in staining are VSMCs. **(B)** Histograms showing the ratio of EdU-positive cells to total cells. **(C)** The MTT assay was used to evaluate the viability of VSMCs. Data are presented as the mean ± S.E.M. ^**^Significant difference compared to the control group at *p* < 0.01, ^##^Significant difference compared to the PDGF-BB group at *p* < 0.01.

To investigate the growth inhibition effect of osthole, cell proliferation was determined by MTT and EdU staining after treatment with osthole at different concentrations. According to images of the EdU assay (Figures 3A,B), the proliferation and viability of VSMCs cultured with PDGF-BB were increased compared with those of the control group (*p* < 0.01), while the proliferation and viability of VSMCs in the 20, 40, and 80μM osthole-treated groups were decreased (*p* < 0.01). As shown in Figure 3C, when VSMCs were treated with DMSO for 24h in the absence of PDGF-BB or osthole, no significant difference was observed in MTT absorbance (*p* > 0.05), suggesting that DMSO was not toxic to the cells. However, the addition of PDGF-BB (25ng/ml) caused a strong increase in VSMC proliferation compared to that of the control group (*p* < 0.01); this increase was inhibited by the addition of osthole at 40 and 80μM (*p* < 0.01).

### Effect of Osthole on VSMC Cell Cycle Progression in the Presence of PDGF-BB

**Figure 4:**
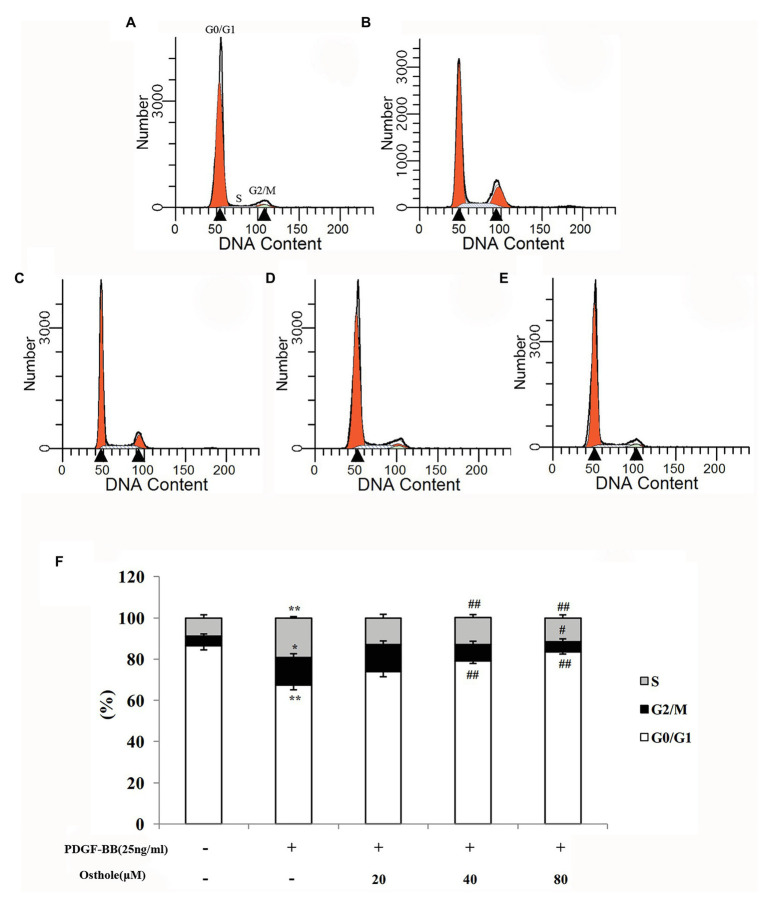
Distribution of cell cycle stages of VSMCs as determined by flow cytometry. Representative images of **(A)** the control group, **(B)** the PDGF-BB group, **(C)** the Osthole-20 group, **(D)** the Osthole-40 group, and **(E)** the Osthole-80 group. **(F)** Percentage of cells in each phase of the cell cycle. Data are expressed as the mean ± S.E.M. *n* = 5~7. In **(F)**, ^*^p < 0.05, ^**^p < 0.01 vs. the control group; ^#^*p* < 0.05, ^##^*p* < 0.01 vs. the PDGF-BB group.

To determine whether osthole influences the cell cycle machinery, we examined cell cycle progression using flow cytometry. As shown in Figures 4A,B,F, cells subjected to a 24-h exposure to PDGF-BB had a significantly higher percentage of cells in and past S phase (*p* < 0.01). After treatment with osthole, most of the VSMCs were arrested at G_0_/G_1_ phase (Figures 4C–F). The PDGF-BB-stimulated VSMCs treated with osthole (20, 40, and 80μM) showed that cell cycle progression was halted at G_0_/G_1_ phase (20μM osthole: G_0_/G_1_ = 73.97%, *p* > 0.05; 40μM osthole: G_0_/G_1_ = 78.9%, *p* < 0.01; 80μM osthole: G_0_/G_1_ = 83.41%, *p* < 0.01).

### Effect of Osthole on the mRNA and Protein Expression of Cyclin D1/CDK4 in VSMCs

**Figure 5:**
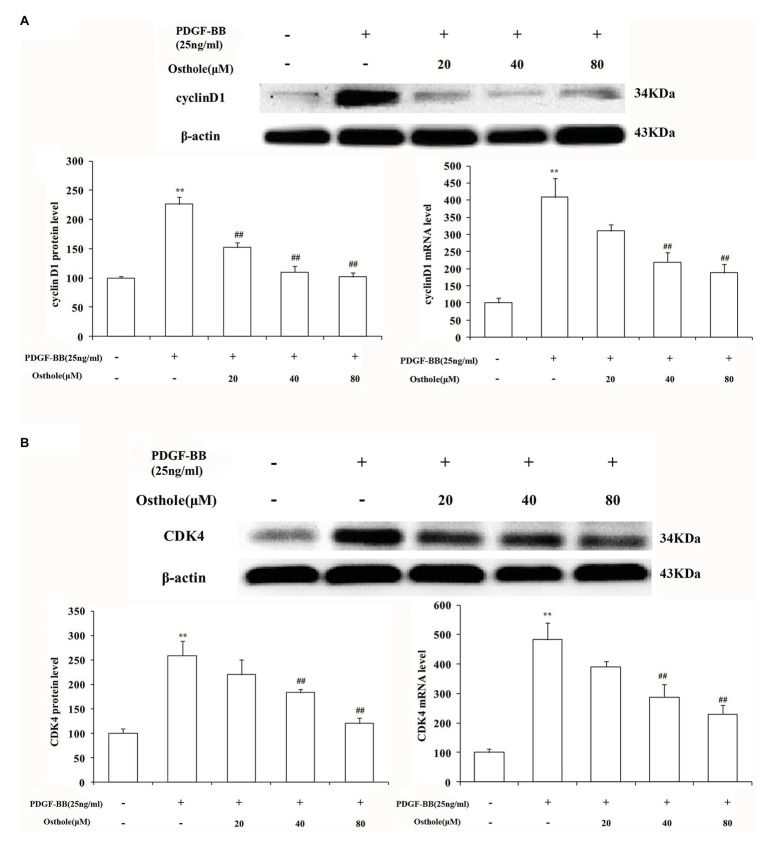
Effect of osthole on cyclin D1/CDK4 mRNA and protein expression in VSMCs. **(A)** Relative mRNA and protein expression of cyclin D1 in VSMCs. **(B)** Relative mRNA and protein expression of CDK4 in VSMCs. Data are expressed as the mean ± S.E.M. *n* = 5. ^**^Significant difference compared to the control group at *p* < 0.01, ^##^Significant difference compared to the PDGF-BB group at *p* < 0.01.

Previous studies have demonstrated that cell cycle progression is tightly regulated by cyclin-CDK complexes during G1 phase. The cyclin D1/CDK4 complex, one such complex, plays a vital role in this process. To determine whether the antiproliferative effect of osthole was associated with the activity of the cyclin D1/CDK4 complex, we analyzed the cyclin D1/CDK4 mRNA and protein expression levels in cultured rat VSMCs using real-time RT-PCR and western blotting, respectively. As expected, our data showed that PDGF-BB (25ng/ml) could elevate the cyclin D1 mRNA and protein expression levels by approximately 4.1-fold and 2.3-fold compared to the levels in the control group, respectively (*p* < 0.01), and osthole could significantly blunt the increases in cyclin D1 mRNA and protein expression (Figure 5A). Meanwhile, cells stimulated with PDGF-BB showed a profound increase in CDK4 mRNA and protein expression compared to the respective levels in the control group (*p* < 0.01). However, treatment with osthole (40 and 80μM) significantly decreased the mRNA and protein expression of CDK4 in PDGF-BB-stimulated VSMCs (*p* < 0.01; Figure 5B).

### Effect of Osthole on the mRNA and Protein Expression of Cyclin E1/CDK2 in VSMCs

**Figure 6:**
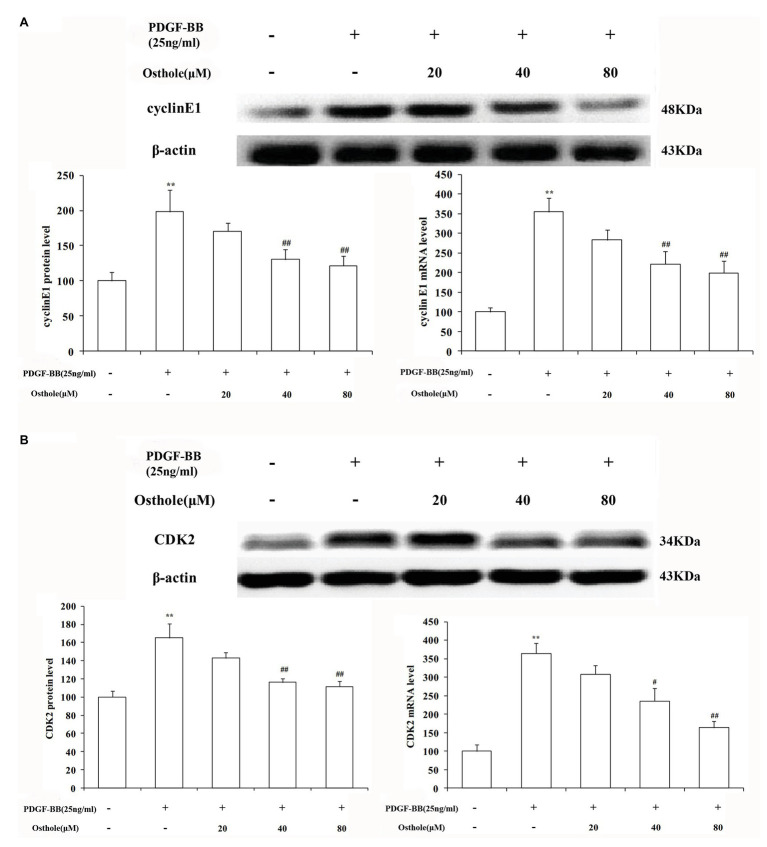
Effect of osthole on cyclin E1/CDK2 mRNA and protein expression in VSMCs. **(A)** Relative mRNA and protein expression of cyclin E1 in VSMCs. **(B)** Relative mRNA and protein expression of CDK2 in VSMCs. Data are expressed as the mean ± S.E.M. *n* = 5. ^**^Significant difference from the control group at *p* < 0.01, ^#^Significant difference from the PDGF-BB group at *p* < 0.05, ^##^Significant difference from the PDGF-BB group at *p* < 0.01.

To further verify our results, we sequentially applied real-time RT-PCR and western blotting to examine the expression levels of cyclin E1/CDK2 mRNA and protein in VSMCs. The results showed that after stimulation with PDGF-BB (25ng/ml), the expression levels of cyclin E1/CDK2 mRNA and protein in VSMCs were markedly increased (*p* < 0.01). After treatment with osthole, we observed an expected decrease in cyclin E1/CDK2 mRNA and protein expression, especially at doses of 40 and 80μM (*p* < 0.01; Figures 6A,B).

## Discussion

Osthole, a traditional Chinese medicine, is now being manufactured in many countries on a large scale with standardized quality. Previous studies have shown that osthole exerts cardiovascular protective activities. Abnormal VSMC proliferation is a major component of cardiovascular disease including atherosclerosis, vein graft occlusion, and restenosis after angioplasty, our *in vivo* experiment suggests that osthole treatment selectively inhibits the proliferation of those VSMC by suppressing inflammation (Wang et al., 2013; Li et al., 2017), Guh’s studies indicated that the antiproliferative effect of osthole occurs early in G1 phase of the VSMC cell cycle and is due to increases in cyclic AMP and cyclic GMP levels (Guh et al., 1996; PMID: 8867108). In this research, we intend to explore whether the molecular mechanisms of osthole on vascular stenosis and VSMC proliferation are related to cell cycle and cell cycle regulatory proteins cyclin D1/CDK4 and cyclin E1/CDK2. We first established the balloon-induced carotid artery injury model in male Sprague-Dawley rats and observed the expression of PCNA, which is used as a marker of DNA synthesis or proliferation, in balloon-treated rat carotid arteries using western blotting, which further confirmed the protective effect of osthole on injured artery walls. We found that VSMC proliferation was proportional to the degree of intimal hyperplasia and that osthole significantly alleviated neointimal hyperplasia in balloon-injured artery walls by inhibiting the proliferation of VSMCs. The results indicated that the changes in PCNA protein expression were very consistent with the histomorphometric changes in the carotid artery walls.

*In vivo*, this dynamic and cyclic process generally begins with a primary regulator such as interleukins (Alexander and Khalil, 2009), interferons (Boshuizen and Winther, 2015), platelet-derived growth factor (PDGF)-BB (Williams et al., 2012), and basic fibroblast growth factor (bFGF; Kesavan et al., 2013), among which PDGF-BB has been considered as the most potent chemoattractant and a strong mitogen present in serum that stimulates DNA synthesis. PDGF-BB can bind to all PDGF receptors, phosphorylate receptors, activate downstream signaling molecules and promotes phenotypic transformation, proliferation, and migration of VSMCs. The importance of PDGF-BB in the development of neointima formation has been established in models of arterial injury (Raines, 2004; Huang et al., 2012; Zhang et al., 2014; Zhao et al., 2014; Fang et al., 2020). Here, we used 25% PDGF-BB as a prototypical mitogen to establish a VSMC proliferation system, we then further examined the role of the osthole on VSMC proliferation *in vitro* experiments. Our data confirmed PDGF-BB induced significant increases in VSMC proliferation, while osthole exhibited a marked antiproliferative effect in VSMCs based on the results of the MTT assay and EdU staining.

Several observations have shown that regulating the cell cycle is an important mechanism for controlling cell proliferation (Liu et al., 2009; Yoo et al., 2013), as entry and the progression of cells through different stages of the cell cycle is an ordered, tightly regulated process involving a complex cascade of events, and cell cycle arrest can trigger the inhibition of proliferation (Eymin and Gazzeri, 2010; Yan et al., 2015). In the present study, cell cycle progression in each group was investigated by flow cytometry, and our results revealed that osthole suppressed PDGF-BB-induced VSMC proliferation and blocked PDGF-BB-induced cell cycle progression of VSMCs from G1 to S phase, which was exhibited by a significant accumulation of VSMCs in G0/G1 phase and a reduction of cells in G2/M+S phases, indicating that osthole-mediated inhibition of VSMC proliferation was attributed to apparent cell cycle arrest quite early during G0/G1 phase.

Cell cycle progression requires different signaling molecules to function at the right time, and the cyclin/CDK complexes play a prominent role because their expression is responsible for the transition from G1 to S phase (Bond et al., 2006; Dong et al., 2016). Both damaged and activated VSMCs induce cyclin D1 expression and thus facilitate the formation of the cyclin D1-CDK4 complex, which is essential for the entry of cells from G0 to G1 phase and prepares cells for the G1/S phase transition. The cyclin D1/CDK4 complex is required to promote the phosphorylation of retinoblastoma (Rb) protein and the exposure of the binding site of E2F1-3 (Liu et al., 2015). Because our studies demonstrated that the treatment of PDGF-BB-stimulated VSMCs with osthole resulted in cell cycle arrest at G1 phase (Figure 4), we then analyzed the expression of cyclin D1/CDK4 to determine whether its effect is due to the downregulation of operative regulatory molecules. Consistent with relevant reported literature (Ge et al., 2016; Chen et al., 2017), high mRNA and protein expression levels of cyclin D1 and CDK4 were found after treatment with PDGF-BB. However, administration of osthole reduced these increases, which were accompanied by inhibition of cell proliferation and cell cycle, suggesting that decreasing cyclin D1/CDK4 expression might be the molecular mechanism by which osthole inhibits VSMC proliferation and arrests the cell cycle.

Furthermore, a growing body of evidence has demonstrated that increased cyclin E1 1 levels are associated with malignancies, such as ovarian cancer (Guo et al., 2011), and elevated cyclin E1 expression is a poor prognostic factor in lung adenocarcinoma patients (Eymin and Gazzeri, 2010). Activated cyclin E1 can bind to CDK2 to form the cyclin E1/CDK2 complex, which also serves to promote DNA replication and further phosphorylates pRb, leading to the formation of a positive feedback loop and regulation of the onset of DNA-synthesis referred to as S-phase (Liu et al., 2015). Interestingly, our results indicate that osthole administration inhibited PDGF-BB-induced cyclin E1-CDK2 upregulation, providing the further evidence for the above studies.

Of note, there still remains a limitation in this study. Although osthole has been proved to prevent cell cycle progression, the target that osthole could bind to is unknown at present, and it is worth further exploration in our next research.

**Figure 7:**
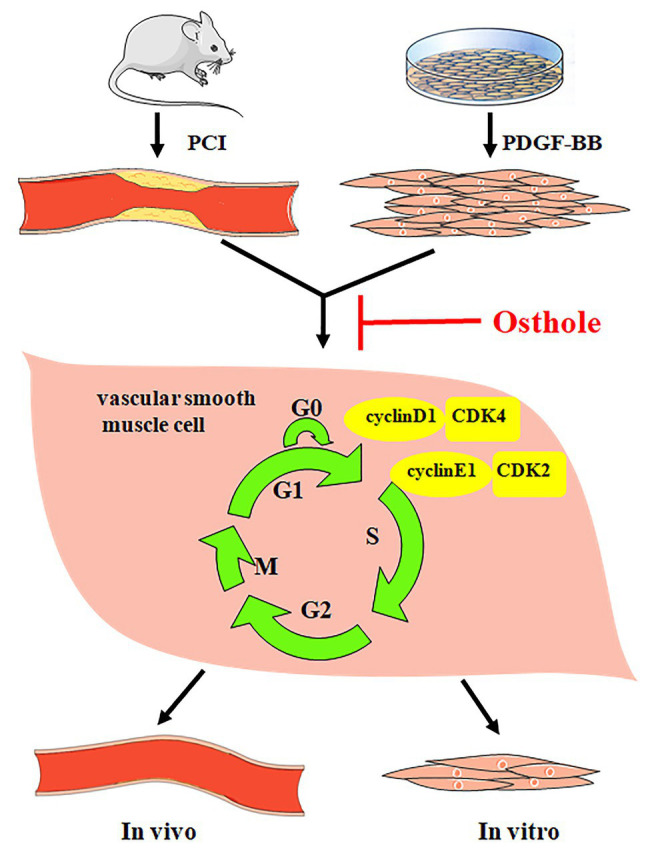
Osthole alleviates neointimal hyperplasia in balloon-induced arterial wall injury and inhibits PDGF-BB-induced VSMC proliferation, and the mechanisms are related to the downregulation of cyclin D1/CDK4 and cyclin E1/CDK2 expression as well as the prevention of cell cycle progression from G0/G1 phase to S phase.

Taken together, our results demonstrate that osthole can significantly alleviate neointimal hyperplasia in balloon-induced arterial wall injury and inhibit PDGF-BB-induced VSMC proliferation, and its regulatory activities are related, at least partially, to downregulation of cyclin D1/CDK4 and cyclin E1/CDK2 expression as well as the prevention of cell cycle progression from G0/G1 phase to S phase (Figure 7).

## Data Availability Statement

All datasets generated for this study are included in the article/supplementary material.

## Ethics Statement

The animal study was reviewed and approved by the Animal Use and Care Committee of Zunyi Medical University.

## Author Contributions

D-LY participated in the study design. D-LY, Y-QL, Y-LL, YG, W-NL, X-TL, and J-YL carried out the experiment *in vivo* and *in vitro*. Q-HG analyzed the results. D-LY and Y-QL prepared the manuscript. All authors contributed to the article and approved the submitted version.

### Conflict of Interest

The authors declare that the research was conducted in the absence of any commercial or financial relationships that could be construed as a potential conflict of interest.
